# Development of Traumatic Neuromas in a Patient Following Endovenous Laser Ablation and Microphlebectomy Procedures: A Rare Complication From the Removal of Varicose Veins

**DOI:** 10.7759/cureus.15830

**Published:** 2021-06-22

**Authors:** Arjun Ahuja, Thomas Grant, Gregory Dumanian, Scott A Resnick

**Affiliations:** 1 Radiology, Chicago Medical School at Rosalind Franklin University of Medicine and Science, North Chicago, USA; 2 Diagnostic Radiology, Northwestern University Feinberg School of Medicine, Chicago, USA; 3 Plastic Surgery, Northwestern University Feinberg School of Medicine, Chicago, USA; 4 Interventional Radiology, Northwestern University Feinberg School of Medicine, Chicago, USA

**Keywords:** neuroma, microphlebectomy, endovenous laser ablation, venous stasis, varicose vein surgery, venous insufficiency, ultrasound (u/s)

## Abstract

Chronic venous insufficiency is one of the most common benign diseases in America. For treatment, minimally invasive techniques have become the first-line option. The literature shows that these procedures are well tolerated and work effectively without leaving the patient with unaesthetic operative scars. We discuss the case of a patient who developed two right lower extremity neuromas as a rare complication following endovenous laser ablation and microphlebectomy procedures for the treatment of varicose veins. Ultrasound is the preferred imaging modality for the visualization and diagnosis of a neuroma and should be performed in post-phlebectomy patients with severe and persistent sensory pattern disruption as neuroma formation can lead to significant complications for the patient.

## Introduction

Chronic venous insufficiency is one of the most common benign diseases in America [1]. It is a condition characterized by inadequate venous valves in the lower extremity that causes poor blood return, stasis, and retrograde flow, leading to a buildup of blood in certain areas of the affected vein. For treatment, minimally invasive techniques, such as endovenous laser ablation (EVLA) and microphlebectomy, have truly become first-rate and high-quality approaches. The literature shows that these procedures are well tolerated and work effectively without leaving the patient with unaesthetic operative scars [2].

After a comprehensive review of the literature, we could only find a single report of a traumatic neuroma developing following an ambulatory surgical phlebectomy in 1994 [3]. The patient had no recurrence or further complications after complete excision of the mass [3]. Here, we present the case of a patient who developed two right lower extremity neuromas as a rare complication following EVLA and microphlebectomy procedures and describe the complicated clinical course that followed.

## Case presentation

The patient was a 45-year-old woman with a history of non-insulin-dependent diabetes mellitus and lower extremity varicose veins. She presented with bilateral great saphenous vein (GSV) and anterolateral saphenous vein reflux with GSV varicosities and symptoms of venous insufficiency. She was initially started on a trial of conservative therapy, including class 2 compression stockings, leg elevation, over-the-counter analgesia, regular exercise, and avoidance of prolonged standing. After unremitting pain that continued to interfere with her activities of daily living, EVLA and microphlebectomy were performed. She underwent laser ablation of the right GSV and the right anterolateral branch of the saphenous vein. Tumescent anesthesia was added with multiple injections along the length of the vein, which ensured that all segments were at least 1 cm deep to the surface of the skin. This insulated the treated vein from the surrounding structures to help prevent thermal injury to the adjacent tissues and skin [1].

**Figure 1:**
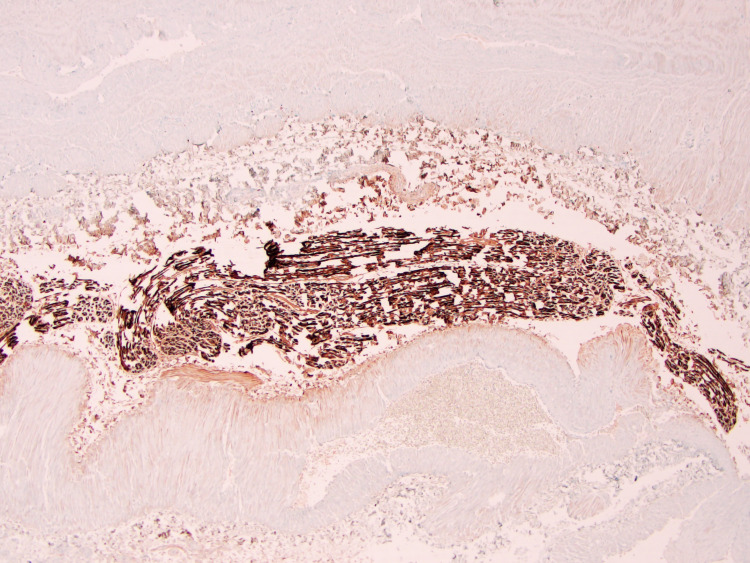
Pathology slide of the removed varicose vein specimen using S100 immunostaining to show the presence of nervous tissue among vessels.

Subsequently, the patient underwent microphlebectomy of 27 veins on the left leg and 21 veins on the right leg. Both sides had extraction sites located from the mid-calf up to the thigh, with an additional extraction site located further distally on the right. The surgical pathology report of the submitted vein specimen showed a segment of a large caliber nerve along with vascular tissue, consistent with varicose veins (Figure 1).

At a one-week routine post-procedure follow-up visit, the patient complained of an area of insensate skin in the dorsum of her foot and the anterior aspect of her lower right leg. She denied any paresthesias or pain. This was seen as a likely consequence of injury to a sensory nerve along the course of one of the removed varicosities.

**Figure 2:**
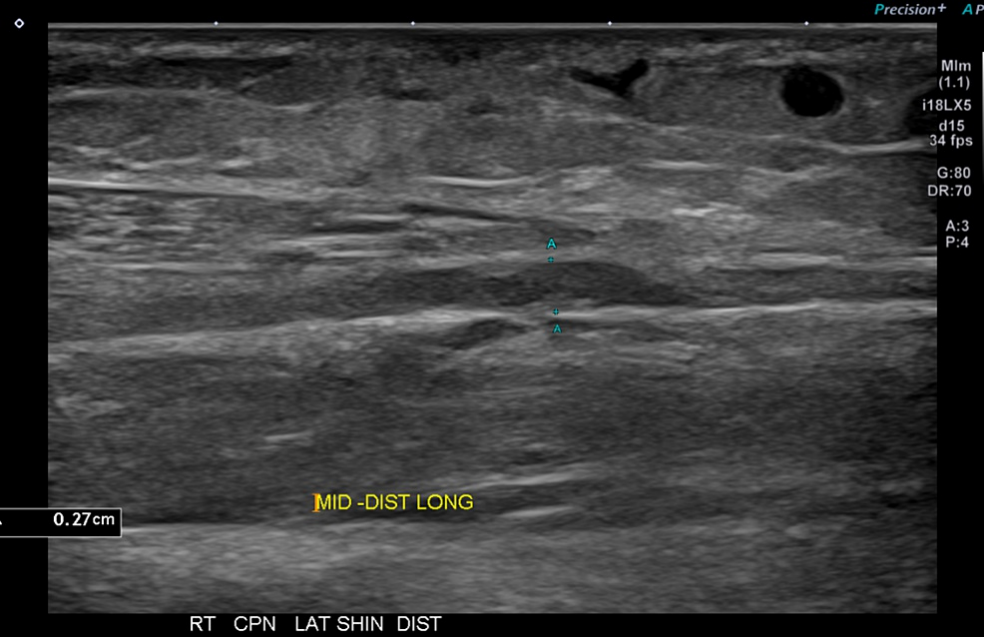
Longitudinal ultrasound image showing a neuroma-in-continuity of the thick superficial branch of the right common peroneal nerve.

The area of anesthesia in her right foot and leg soon developed a burning sensation. The lack of feeling with associated dysesthesias was located in the superficial peroneal nerve distribution. In addition, the patient complained of an additional area complicated by a mild tingling sensation located medial to her right knee. Her other leg was without any symptoms. On ultrasound, in the right mid-calf, the superficial branch of the common peroneal nerve demonstrated focal thickening consistent with a neuroma (Figure 2).

**Figure 3:**
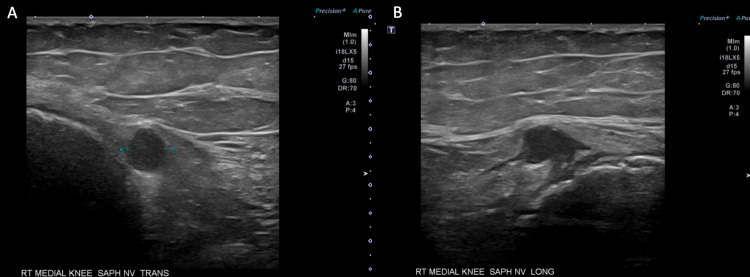
Ultrasound images showing a neuroma of the patellar branch of the right saphenous nerve adjacent to the knee. A: Transverse view. B: Longitudinal view.

Additionally, near one of the extraction sites immediately adjacent to the right medial patella, there was a 1.2 cm soft tissue mass without vascular flow with nerve coursing through, which was felt to indicate a traumatic neuroma of the patellar branch of the saphenous nerve (Figure 3).

Since the patient had a significant amount of pain where it was presumed the superficial peroneal nerve neuroma was located, along with significant dysesthesias in her right lower leg and foot, surgical exploration was performed in that area. Direct visualization showed that under the most caudal phlebectomy incision site on the right, the superficial peroneal nerve remained in continuity, but appeared scarred and neuromatous. Further down the leg, the nerve fascicles appeared normal again. The abnormal segment was approximately 7 cm in length. The superficial peroneal nerve neuroma-in-continuity was resected, and an autograft from the deep peroneal nerve was sewn to reconstruct the nerve gap. The deep peroneal nerve was then divided mid-calf, and targeted muscle reinnervation (TMR) was performed using the proximal end of the deep peroneal nerve, which was transferred to a newly divided motor point of the extensor hallucis longus muscle. The surgical pathology report of the resected superficial peroneal nerve neuroma showed a segment of the nerve with focal myxoid change, measuring 6.5 × 1.1 × 0.3 cm.

At follow-up visits months after her initial procedures, the patient was still not ambulating well, which she attributed to the persistent pain. On examination, there was no apparent foot drop and her dorsiflexors and plantar flexors were still functioning, albeit with a reduced range of motion. Even after treatment with 50 mg of tramadol, as needed, every six hours, 75 mg of pregabalin three times daily, lidocaine patches, acetaminophen, and ibuprofen, she remained in pain and stayed hesitant to move her foot. In addition, she had not been able to return to work since her initial EVLA and microphlebectomy procedures. To test if the patient’s continued symptoms were due to persistent irritation of the nerve and whether the reduced range of motion around the ankle was due to pain itself, 6 mL of Xylocaine with epinephrine was injected to anesthetize the nerve. After this, she walked naturally and was able to move her foot with a full range of motion. Due to the definite improvement of function with pain relief, a superficial peroneal nerve excision and TMR were recommended to the patient, even though it would leave her numb on her foot for the rest of her life. As medication failed to reduce her symptoms, a nerve excision would at least ease the pain stemming from her irritated superficial peroneal nerve, which was shown to be causing her ambulatory difficulties.

## Discussion

For the treatment of varicose veins, minimally invasive techniques such as EVLA and microphlebectomy have been shown to work effectively while causing minimal complications for patients. EVLA uses a laser to achieve thermal ablation of the designated vein. Microphlebectomy, also known as ambulatory phlebectomy, involves making several 2-3 mm incisions over the bulging varices and then using a small vein hook coupled with fine clamps to excise the veins in question [4]. The most commonly recognized complications include superficial burns, arteriovenous fistulas, deep vein thromboses, infections, hematomas, nerve damage, as well as pain and bruising [4,5]. Nerve injuries occur due to the proximity of various nerves to the ablated or removed veins. Based on previous literature reports, damage to these nerves generally only causes transient cutaneous paresthesias that largely resolve within weeks to months [5]. The formation of a neuroma following these procedures is a very rare occurrence.

Neuromas are benign neural masses that typically form in response to peripheral nerve injury as a result of excess or irregular hyperplasia [6-8]. Pathologically, any nerve that is lacerated, avulsed, or traumatized can potentially form a neuroma [6]. They are generally classified into two broad categories: neuroma-in-continuity or an end-bulb/stump neuroma [7]. A neuroma-in-continuity forms when the proximal and distal nerve fibers at the site of injury fail to join. This can be due to surrounding framework disruption, disorganized regeneration, hypertrophy of nerve fascicles, or surrounding fibrosis [6]. End-bulb neuromas can develop anywhere a nerve is fully severed and unimpeded by other neural tissue. These can be further classified as a neuroma in a completely severed nerve and an amputation neuroma. They can occur in lacerations where the nerve is not repaired promptly, in amputation stumps, and in postoperative patients where nerves may have been divided accidentally [6]. Histologically, neuromas are characterized as a non-encapsulated, non-neoplastic mass of Schwann cells, endoneurial cells, perineurial cells, and axons embedded in a dense fibrous matrix [7]. Additionally, painful neuromas are characterized by histological signs of chronic inflammation such as inflammatory molecules and myofibroblasts [8].

## Conclusions

In this report, we describe the development of two traumatic neuromas in a patient after undergoing both EVLA and microphlebectomy procedures for the treatment of varicose veins. Even after complete excision of the most symptomatic neuroma of her right superficial peroneal nerve, she remained in pain and was unable to return to her baseline level of functioning. While nerve injury is a known potential complication following common procedures used for the removal and treatment of varicose veins, the formation of a traumatic neuroma is rare and can be significantly debilitating for the patient, as evidenced by this case report. After a comprehensive review of the literature, we could only find a single previous case illustrating the formation of a traumatic neuroma following an ambulatory phlebectomy, with very little information describing the subsequent procedural undertakings for the possible complex clinical course that can ensue. We wanted to further demonstrate that neuroma formation can be a significant potential complication of microphlebectomy (ambulatory phlebectomy) and should be considered in post-phlebectomy patients with severe and persistent sensory pattern disruption. Ultrasound is the preferred imaging modality for the visualization and diagnosis of a neuroma. Further, although not always successful in restoring the baseline neurologic and functional status, surgical options for neuroma treatment exist.
